# α-bisabolol Is an Effective Proapoptotic Agent against BCR-ABL^+^ Cells in Synergism with Imatinib and Nilotinib

**DOI:** 10.1371/journal.pone.0046674

**Published:** 2012-10-03

**Authors:** Massimiliano Bonifacio, Antonella Rigo, Emanuele Guardalben, Christian Bergamini, Elisabetta Cavalieri, Romana Fato, Giovanni Pizzolo, Hisanori Suzuki, Fabrizio Vinante

**Affiliations:** 1 Department of Medicine, Section of Hematology, University of Verona, Verona, Italy; 2 Department of Biochemistry “G. Moruzzi”, University of Bologna, Bologna, Italy; 3 Department of Life and Reproduction Sciences, Section of Biochemistry, University of Verona, Verona, Italy; University of Queensland, Australia

## Abstract

We showed that α-bisabolol is active against primary acute leukemia cells, including BCR-ABL^+^ acute lymphoblastic leukemias (ALL). Here we studied the activity of α-bisabolol against BCR-ABL^+^ cells using 3 cell lines (K562, LAMA-84, CML-T1) and 10 primary BCR-ABL^+^ ALL samples. We found that: (a) α-bisabolol was effective in reducing BCR-ABL^+^ cell viabilty at concentrations ranging from 53 to 73 µM; (b) α-bisabolol concentrations in BCR-ABL^+^ cellular compartments were 4- to 12-fold higher than in normal cells, thus indicating a preferential intake in neoplastic cells; (c) α-bisabolol displayed a slight to strong synergism with the Tyrosine Kinase Inhibitors (TKI) imatinib and nilotinib: the combination of α-bisabolol+imatinib allowed a dose reduction of each compound up to 7.2 and 9.4-fold respectively, while the combination of α-bisabolol+nilotinib up to 6.7 and 5-fold respectively; (d) α-bisabolol-induced apoptosis was associated with loss of plasma membrane integrity, irreversible opening of mitochondrial transition pore, disruption of mitochondrial potential, inhibition of oxygen consumption and increase of intracellular reactive oxygen species. These data indicate α-bisabolol as a candidate for treatment of BCR-ABL^+^ leukemias to overcome resistance to TKI alone and to target leukemic cells through BCR-ABL-independent pathways.

## Introduction

The tyrosine kinase inhibitors (TKI), such as imatinib, dasatinib and nilotinib, have impressively changed the outcome of BCR-ABL^+^ leukemias by targeting and silencing the BCR-ABL kinase. To date, treatment with TKI entails high rates of durable complete cytogenetic and molecular responses, particularly in chronic myeloid leukemia (CML) in chronic phase. However, about 25–30% of patients develop resistance or intolerance to imatinib and only a minority of treated individuals remain disease free after therapy discontinuation, thus indicating that TKI do not eradicate the primitive BCR-ABL^+^ leukemic stem cells [1]–[4]. Therefore, a number of studies have addressed the question if different anti-cancer compounds could display a therapeutic efficacy in combination with TKI: among others, standard chemotherapy [5], and inhibitors of serine/threonine kinase [6], farnesyl transferase [7], proteasome [8], hedgehog pathway [9], or histone deacetylase [10] have been tested both *in vitro* and *in vivo*. Collectively, these studies raise the prospect that rationally-designed combination therapies including non-TKI and TKI compounds may further improve the outcome of BCR-ABL^+^ leukemias.

α-bisabolol is a small, plant-derived, oily sesquiterpene alcohol with some anti-inflammatory and even anti-microbial properties [11]. We discovered that α-bisabolol exerts a selective pro-apoptotic action towards human malignant cells, both non-hematological and leukemic. In an *in vitro* model of glioblastoma cell lines α-bisabolol induced apoptosis through the mitochondrial pathway, by abolishing the mitochondrial transmembrane potential (ΔΨ_m_) and inducing the release of cytochrome *c* [12]. We showed that α-bisabolol exerted a pro-apoptotic activity in an *ex vivo* leukemic model through a similar mechanism [13]. α-bisabolol may induce preferential toxicity against tumor cells because it enters the cells through lipid rafts [14], that are more represented in tumor cells than their normal counterparts [15]. The specific intracellular target of α-bisabolol has not been defined yet: structural similarities suggest that α-bisabolol could be able to interact with BH3-only domain proteins. These mediate activation of the mitochondrial transition permeability pore (mPTP), whose irreversible opening leads to ΔΨ_m_ dissipation, subsequent activation of caspases and execution of apoptosis [14], [16]–[17]. Also BH3-only proteins control the initiation of the autophagic process [18].

In the present study, we determined the activity of α-bisabolol against BCR-ABL^+^ cell lines and primary cells and investigated the molecular mechanism by which α-bisabolol induced apoptosis in these cells. We demonstrate that α-bisabolol synergistically enhances the apoptotic effects of imatinib and nilotinib in BCR-ABL^+^ cells, through induction of mitochondrial membrane damage, at least partially *via* mPTP activation and irreversible opening. The use of drug combination allows to reduce imatinib and nilotinib up to 9-fold to obtain the same cytotoxic effect. These findings suggest that α-bisabolol and TKI could represent a viable combination treatment for BCR-ABL^+^ leukemias, potentiating the efficacy or allowing the dose reduction of TKI.

## Materials and Methods

### Cells and Ethical Requirements

#### 1. Cell lines

The imatinib and nilotinib-sensitive BCR/ABL^+^ K562, LAMA-84 and CML-T1 cell lines (blast crisis of human chronic myeloid leukemia, purchased from DSMZ, Braunschweig, DE) were used in this study.

#### 2. Primary leukemic cells

Viable leukemic cells of 10 patients with untreated BCR-ABL^+^ Acute Lymphoblastic Leukemia (ALL) were purified as previously described [19] on a Ficoll-Hypaque gradient either from peripheral blood in case of a circulating blast count ≥30,000/µL, or from full-substituted bone marrow that was frozen in liquid nitrogen at diagnosis. In all cases cell viability at thawing was >90%.

#### 3. Normal peripheral blood mononuclear cells (PBMC)

Normal PBMC were collected from freshly heparinized peripheral blood of 5 healthy donors. Mononuclear cells were separated on a Ficoll-Hypaque gradient and used in parallel with cell lines for cytotoxicity assays and for measurement of α-bisabolol concentration in cellular fractions. A written informed consent was obtained from ALL patients and from healthy volunteers, according to Italian law. This study was approved by the ethics committee of the Verona University Hospital.

### Cytotoxicity Assays

Cells resuspended in RPMI-1640 (Invitrogen, Carlsbad, CA), supplemented with 10% heat-inactivated fetal bovine serum (Invitrogen), 50 U/mL penicillin and 50 µg/mL streptomycin (complete medium, CM), seeded at a density of 2×10^4^ cell/mL in 96-well plates and incubated at 37°C in 5% CO_2_ were exposed for 48 hours to incremental concentrations of α-bisabolol (dissolved in ethanol 1∶8; Sigma-Aldrich, St. Louis, MO) to determine the half maximal inhibitory concentration (IC_50_) for each cell population. Cytotoxicity was measured by 3-(4,5-dimethylthiazol-2-yl)-2,5-diphenyltetrazolium bromide MTT (Sigma-Aldrich) incorporation as previously described [20], [21] and was expressed as ratio of number of cells treated with α-bisabolol to number of cells treated with vehicle alone. To compare the differential sensitivity to α-bisabolol of blasts vs normal cells, flow cytometry analysis was carried out in three selected BCR-ABL^+^ ALL patients, whose bone marrow samples contained 10 to 20% of residual normal T-lymphocytes. Samples were treated with 20, 40, 80 µM α-bisabolol for 24 hours, then immunostained with anti-CD10 APC, anti-CD3 FITC and anti-CD19 PE (Becton Dickinson, San Jose, CA) monoclonal antibodies (moAbs). At least 5×10^4^ cells of each sample were acquired on a FACSCanto cytometer (Becton Dickinson) and subjected to PolyChromatic Plot analysis by FlowJo 9.3.3 software (Tree Star, Ashland, OR).

### Synergism Studies

BCR-ABL^+^ cell lines and ALL primary samples were treated with α-bisabolol or the TKI imatinib or nilotinib (generous gifts of Novartis, Basel, Switzerland), as single agents or combinations of α-bisabolol and imatinib or α-bisabolol and nilotinib. The concentration of each agent that inhibit half cell viability (IC_50_) was preliminarly determined in cell lines to derive *constant ratio* combination designs. We used α-bisabolol concentrations up to 160 µM for all the three cell lines, imatinib concentrations up to 200, 400, and 800 nM for LAMA-84, CML-T1, and K562 cells, respectively, nilotinib concentrations up to 20 nM for LAMA-84, and up to 40 nM for CML-T1 and K562 cells. Primary cells were treated with concentrations of α-bisabolol from 10 to 160 µM, imatinib from 50 to 800 nM and nilotinib from 5 to 80 nM. Cytotoxicity was evaluated by MTT assay. The effects of interaction between α-bisabolol and TKI were analyzed according to the median-effect method of Chou and Talalay [22] using the CalcuSyn Software (Biosoft, Cambridge, UK). The mean combination index (CI) values were assessed and combination data were depicted as CI vs. fraction affected (Fa) plots, defining the CI variability by the Sequential Deletion Analysis method. CI <1 represented a synergistic effect (<0.1 = very strong synergism; 0.1–0.3 = strong synergism; 0.3–0.7 = synergism; 0.7–0.85 = moderate synergism; 0.85–0.90 = slight synergism). Also dose-reduction index (DRI) for each combination was calculated.

### Cellular Fractionation and α-bisabolol Extraction

To measure α-bisabolol concentrations in cellular fractions, cell lines and normal PBMC were incubated with 40 µM α-bisabolol for 24 hours. Then, cells were washed with PBS, harvested by centrifugation at 200 *g* for 10 minutes, and subcellular fractions were obtained according to Imai et al. [23] with minor modifications. Cytosol and pellet fractions were treated with methanol 1∶3 (v/v) and extracted with exane 1∶1 (v/v). Samples were stirred for 10 minutes and centrifugated at 3000 *g* for 15 minutes, then the exane layers were collected and dried under nitrogen flux. Samples were resuspended in acetonitrile for HPLC analysis, as published [24]. A Waters 510 HPLC system, Kinetex C18 column (100×4.6 mm, 2.6 µm; Phenomenex, Torrance, CA) and Waters 996 Photodiode Array detector were used to perform all chromatographic runs. Calibration curves for α-bisabolol quantification were obtained using freshly prepared standard solutions at concentrations ranging from 0.2 to 5 nM.

### Evaluation of Plasma Membrane Integrity

Cell lines were treated for 1 to 3 hours with 80 µM α-bisabolol, then washed with PBS and stained with Annexin-V-FITC (Miltenyi Biotec, Bergisch Gladbach, DE) for 15 minutes and TO-PRO-3 (Invitrogen) immediately before acquisition on a FACSCalibur cytometer (Becton Dickinson) at a detection wavelength of 525 nm for Annexin V and 661 nm for TO-PRO-3. Treated cells were also incubated with 100 nM DiBAC4(3) [Bis-(1,3-Dibutylbarbituric Acid)Trimethin Oxonol – Invitrogen] for 20 minutes: samples were analyzed by flow cytometry at a detection wavelength of 516 nm. To evaluate Ca^2+^ influx, cells were resuspended in HBSS/Ca^2+^ and loaded with 2 µM Fluo-4 AM (Invitrogen) for 45 minutes at 37°C. Then cells were washed and incubated for 20, 40 and 60 minutes with 80 µM α-bisabolol. Time-dependent cell fluorescence was recorded at a detection wavelength of 516 nm. Data were analyzed using the software FlowJo 9.3.3.

### Evaluation of Mitochondrial Membrane Integrity

Cell lines were resuspended in CM at 1×10^6^/mL and treated with 40 µM α-bisabolol for 3 and 5 hours at 37°C. Evaluation of mitochondrial transmembrane potential (ΔΨ_m_) was performed as previously described [13]. Briefly, cells were washed with pre-warmed CM, loaded with 4 µM JC-1 (5,5′,6,6′-tetra-chloro-1,1′,3,3′-tetra-ethyl-benz-imidazolyl-carbo-cyanine iodide, Molecular Probes, Eugene, OR) and after 30 minutes incubation they were washed twice with PBS. An aliquot of each sample was spotted onto a slide, mounted with a coverslip and immediately recorded by an Axio Observer inverted microscope (Zeiss, Gottingen, DE). Visualization of JC-1 monomers (green fluorescence) and JC-1 aggregates (red fluorescence) was done using filter sets for fluorescein and rhodamine dyes. Image analysis was done using Axiovision 3 software. The other aliquot of each sample was resuspended in PBS and analyzed by flow cytometry. Evaluation of mitochondrial permeability transition pore (mPTP) was done by the MitoProbe Transition Pore assay kit (Invitrogen). Briefly, cells were washed with CM, resuspended with HBSS/Ca^2+^ and loaded with 10 nM Calcein AM with or without 400 µM CoCl_2_ for 15 minutes at 37°C. Cell fluorescence was recorded at 516 nm wavelenght.

### Oxygen Consumption

Intact BCR-ABL^+^ cell lines, in the presence or absence of 40 µM α-bisabolol for 24 hours, were assayed for oxygen consumption at 30°C in DMEM (Invitrogen) using a thermostatically controlled oxygraph and Clark electrode. Endogenous cell respiration was recorded and the maximal respiration rate (uncoupled respiration) was empirically determined by the addition of 500 nM carbonylcyanide-4-(trifluoromethoxy)-phenylhydrazone (FCCP). Oxygen consumption was completely inhibited by adding 4 µM antimycin A at the end of the experiments [25].

### Detection of Intracellular Reactive Oxigen Species (ROS)

Cell lines resuspended in HBSS (Invitrogen) at 5×10^5^/mL were loaded with 2.5 µM of the membrane-permeable probe 5-(and-6)-chloromethyl-2′7′-dichlorodihydrofluorescein diacetate acetyl ester (CM-H_2_DCFDA, Molecular Probes) for 1 hour at 37°C, as previously described [26]. Cells were stimulated with 40 µM α-bisabolol for 5 hours. ROS generation was evaluated in flow cytometry by measuring the green fluorescence signal of DCF, the oxidation product of CM-H_2_DCFDA by free radicals. N-acetylcysteine (NAC) was used at 5 mM concentration as ROS scavenger.

### Statistics

Student’s t-test for means, chi-squared tests and Kruskall-Wallis analysis of variance by rank were considered significant for *p* values *<*0.05.

## Results

### α-bisabolol Inhibits BCR-ABL^+^ Cells Viability

**Figure 1:**
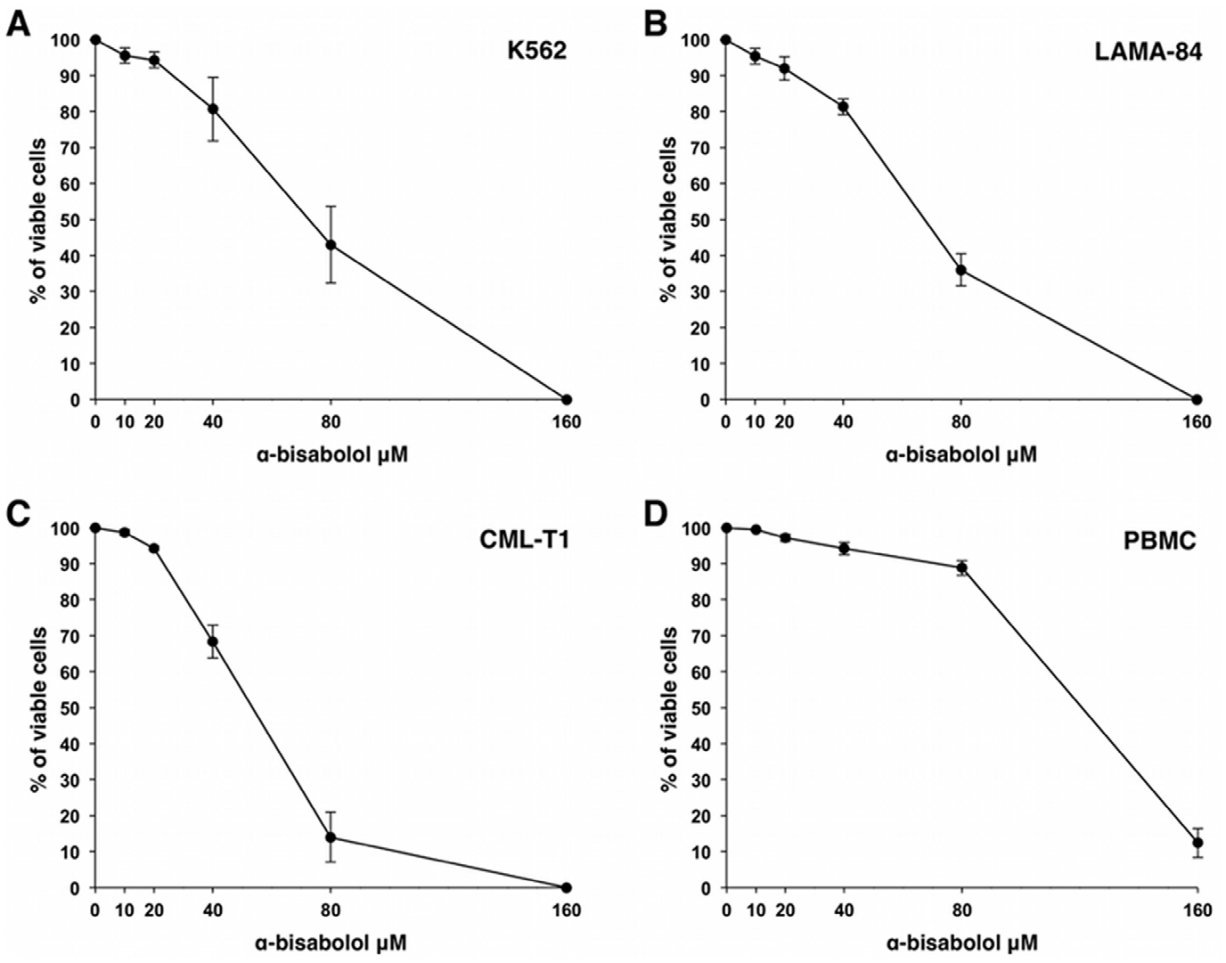
α-bisabolol reduced BCR-ABL^+^ cell viability. (A-C) Cell lines were treated with α-bisabolol at the indicated concentration for 48 hours. Results are expressed as mean±SD of 5 experiments for each cell line. (D) Normal PBMC (n = 5), in the same experimental conditions, are significantly less sensitive to α-bisabolol (p = .03).

**Figure 2:**
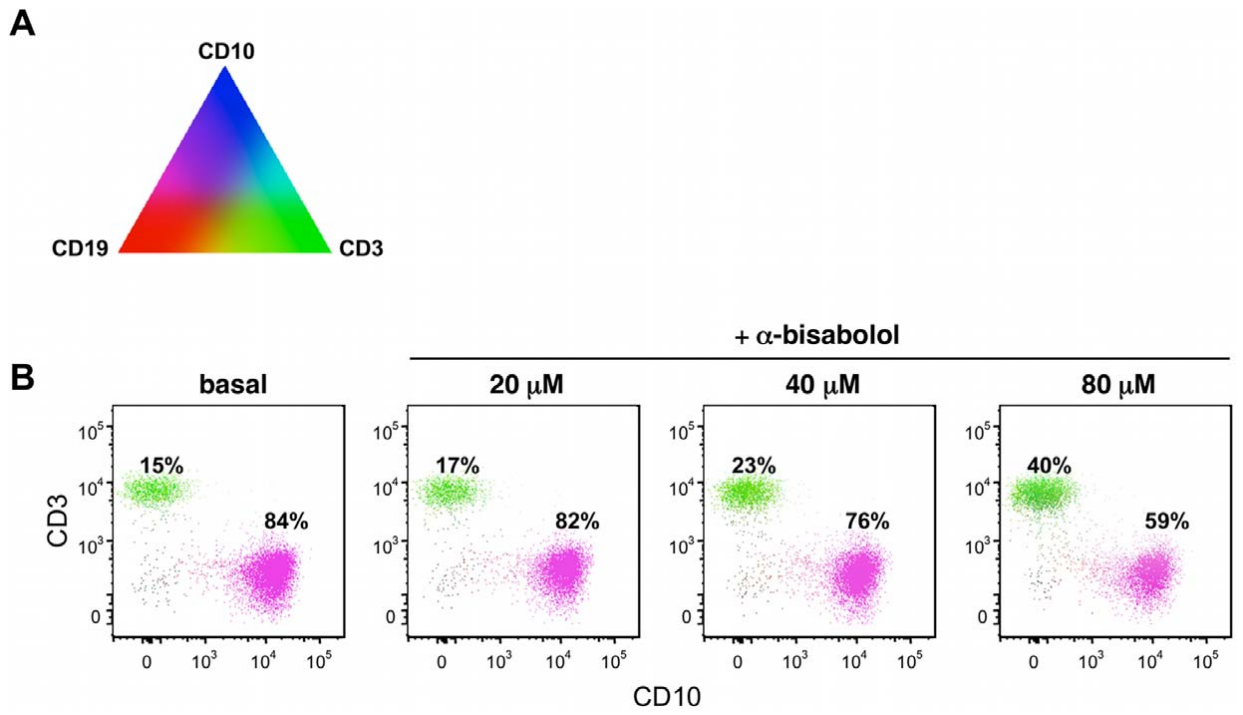
PolyChromatic Plot analysis of specificity of action of α-bisabolol on BCR-ABL^+^ ALL blasts as compared to normal lymphocytes. Bone marrow mononuclear cells of a representative case of BCR-ABL^+^ B-ALL with 15% of T-lymphocytes (patient #08) were treated with 20, 40, 80 μM α-bisabolol for 24 hours, then immunostained with anti-CD3 FITC, anti-CD10 APC and anti-CD19 PE moAbs and analyzed by flow cytometry. (A) The triangle illustrates the different dummy colors assigned to each marker and the mixtures that results by their different amounts. (B) The bivariate PolyChromatic plots show the color mapping result: subsets of cells can be identified by the color scheme. Cells expressing only CD3 are green, those with CD19 and CD10 are magenta (i.e. blue + red). Normal CD19^+^ B-cells (i.e. blue) are about 1% in each plot. The comparison of the plots gives evidence that α-bisabolol induces a dose-dependent preferential depletion of blasts while preserving normal lymphocytes.

Treatment with 10 to 160 µM α-bisabolol for 48 hours resulted in a dose-dependent reduction of BCR-ABL^+^ cell viability by MTT assay. IC_50_ was 53±5, 68±3 and 73±9 µM for CML-T1, LAMA-84 and K562 cells respectively (figure 1°, B, C). In the same experiments we compared the effect of α-bisabolol on viability of normal PBMC, which proved significantly less sensitive to treatment (IC_50_>0.1 mM) than BCR-ABL^+^ cell lines (p = .03; figure 1D). By flow cytometry we also evaluated the differential sensitivity to α-bisabolol of BCR-ABL^+^ primary ALL blasts and normal residual T lymphocytes within the same samples. As shown in figure 2, α-bisabolol induced a preferential depletion of leukemic cells. These data indicate that α-bisabolol is effective in inhibiting BCR-ABL^+^ cell viability in a dose-dependent manner at concentrations that spare normal cells.

### α-bisabolol is Synergistic with Imatinib and Nilotinib in BCR-ABL^+^ Cells

**Figure 3:**
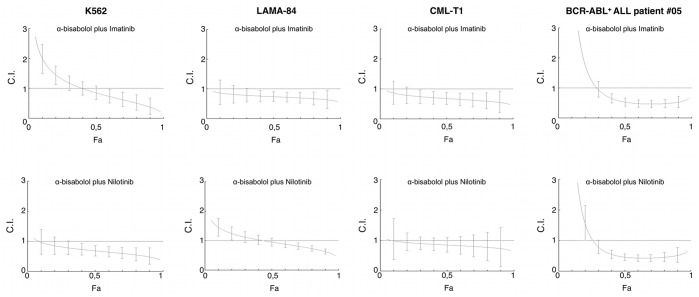
Fa-CI plots of interaction between α-bisabolol and TKIs. α-bisabolol plus imatinib or nilotinib proved to be synergistic both in BCR-ABL^+^ cell lines and primary leukemic cells (CI <0.1 = very strong synergism; 0.1–0.3 = strong synergism; 0.3–0.7 = synergism; 0.7–0.85 = moderate synergism; 0.85–0.90 = slight synergism).

After studying the efficacy of single agent α-bisabolol, we sought to evaluate its properties in combination with established drugs active in BCR-ABL^+^ leukemias, such as TKI imatinib and nilotinib. We combined α-bisabolol and imatinib or nilotinib at constant ratio, according to the median-effect method by Chou and Talalay [22]. As summarized in table 1, the combination of α-bisabolol with imatinib or nilotinib showed slight to strong synergistic effects both in cell lines and in primary BCR-ABL^+^ blasts, except for one single case (patient #03). Figure 3 represents the Fa-CI plots for each cell line and a representative case of BCR-ABL^+^ ALL.

**Table 1:** Combination indices (CI) in synergysm experiments using drugs in constant ratio.

	α-bisabolol – imatinib				α-bisabolol – nilotinib			
	CI at IC_50_	CI at IC_75_	CI at IC_90_	CI at IC_95_	CI at IC_50_	CI at IC_75_	CI at IC_90_	CI at IC_95_
K562	0.850	0.563	0.377	0.289	0.686	0.576	0.484	0.431
LAMA-84	0.741	0.686	0.635	0.603	0.899	0.777	0.635	0.552
CML-T1	0.683	0.613	0.553	0.517	0.858	0.797	0.742	0.707
Pt. #01	0.279	0.118	0.051	0.019	0.457	0.214	0.102	0.061
Pt. #02	0.595	0.32	0.309	0.317	0.28	0.247	0.261	0.269
Pt. #03	1.277	1.111	1.113	1.162	1.571	0.958	0.673	0.602
Pt. #04	0.846	0.559	0.552	0.593	0.436	0.427	0.502	0.561
Pt. #05	0.531	0.473	0.531	0.591	0.46	0.442	0.518	0.587
Pt. #06	0.949	0.627	0.64	0.661	0.842	0.602	0.633	0.663
Pt. #07	0.959	0.768	0.773	0.801	nd	nd	nd	nd
Pt. #08	0.712	0.665	0.611	0.578	0.599	0.543	0.476	0.445
Pt. #09	0.919	0.949	0.983	0.869	0.876	0.887	0.914	0.934
Pt. #10	0.704	0.735	0.807	0.869	0.669	0.727	0.808	0.872

The Dose Reduction Index (DRI) is a measure of how many folds the dose of each drug in a synergistic combination may be reduced at a given effect level when compared with the doses of each drug alone. In table 2 the DRI of drug combinations for concentrations that inhibit 50, 75, 90 and 95 per cent of cell viability (IC_50_, IC_75_, IC_90_, IC_95_ respectively) are reported. The combination of α-bisabolol and imatinib allowed a dose reduction up to 7.2 and 9.4-fold respectively; the combination of α-bisabolol and nilotinib allowed a dose reduction up to 6.7 and 5-fold respectively.

**Table 2:** Dose Reduction indices (DRI) in synergysm experiments using drugs in constant ratio.

	DRI at IC_50_				DRI at IC_75_				DRI at IC_90_				DRI at IC_95_			
	α-bis – imatinib		α-bis – nilotinib		α-bis – imatinib		α-bis – nilotinib		α-bis – imatinib		α-bis – nilotinib		α-bis – imatinib		α-bis – nilotinib	
K562	3.426	1.791	4.257	2.215	4.518	2.924	5.050	2.640	5.959	4.774	5.991	3.147	7.194	6.663	6.729	3.546
LAMA-84	2.416	3.061	3.625	1.471	2.661	3.228	4.145	1.867	2.931	3.403	4.739	2.369	3.129	3.527	5.191	2.786
CML-T1	2.072	4.984	1.714	3.644	2.199	6.314	1.808	4.092	2.335	7.998	1.907	4.956	2.432	9.393	1.977	4.974

### α-bisabolol Preferentially Concentrates in both Membrane/nuclei and Cytosolic Compartments of BCR-ABL^+^ vs Normal Cells

**Figure 4:**
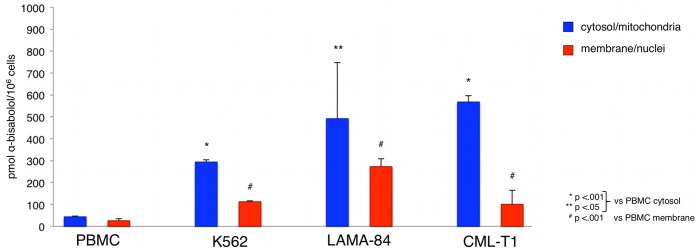
α-bisabolol concentrations in cellular compartments of BCR-ABL^+^ vs normal cells. Cell lines were treated with 40 µM α-bisabolol for 24 hours. α-bisabolol concentrations were significantly higher both in cytosol and membrane/nuclei compartments of BCR-ABL^+^ vs normal cells. The results are expressed as mean±SD of 3 experiments for each cell line.

We have previously demonstrated that α-bisabolol enters cells *via* lipid rafts [14], highly dynamic membrane structures which are far more represented in neoplastic cells than in their normal counterparts [15]. To further study the cellular distribution of α-bisabolol we fractionated BCR-ABL^+^ and normal cells into a supernatant fraction containing cytosol organelles including mitochondria, and a pellet fraction containing nuclei and cellular membranes. By HPLC technique we measured the actual concentrations of α-bisabolol in the cellular compartments after 24 hours incubation with 40 µM α-bisabolol. We found that α-bisabolol concentration in the cytosol of K562, LAMA-84 and CML-T1 cells (294±11, 492±256 and 568±30 pmol/10^6^ cells, respectively) is 6 to 12-fold higher than in normal PBMC (44±4 pmol/10^6^ cells). Similarly, α-bisabolol concentration in the membrane and nuclei cellular fraction of BCR-ABL^+^ cells (113±4, 273±36 and 101±64 pmol/10^6^ cells for K562, LAMA-84 and CML-T1, respectively) is 4 to 10-fold higher than in normal PBMC (26±10 pmol/10^6^ cells, figure 4). These data indicate that α-bisabolol preferentially concentrates in cellular membranes, possibly due to their higher content in lipid rafts, and in the cytosol fraction of BCR-ABL^+^ cells than in the respective compartments of normal cells.

### α-bisabolol Rapidly Determines Loss of Plasma Membrane Integrity in BCR-ABL^+^ Cells

**Figure 5:**
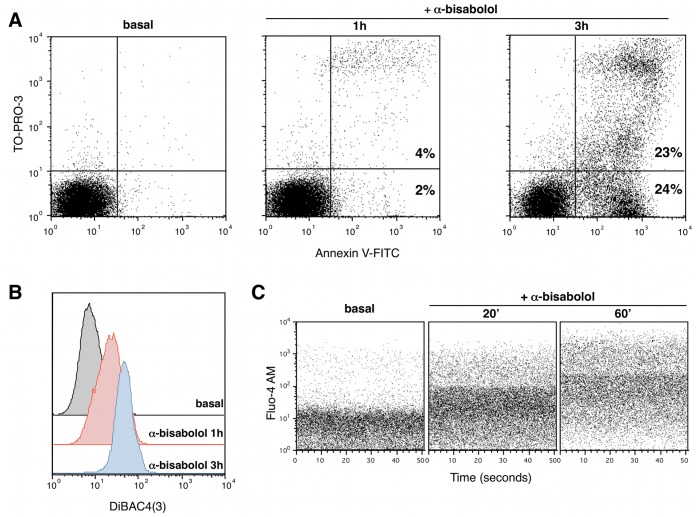
α-bisabolol caused rapid loss of plasma membrane integrity in BCR-ABL^+^ cells (CML-T1 cell line). (A) Cells were treated with 80 µM α-bisabolol for 1 and 3 hours, then stained with Annexin-V and TO-PRO-3 to distinguish between unaffected cells (AnxV^neg^ TO-PRO-3^neg^), early apoptosis (AnxV^pos^ TO-PRO-3^neg^) and late apoptosis (AnxV^pos^ TO-PRO-3^pos^). (B) Time-dependent increase of cell fluorescence after incubation with α-bisabolol and DiBAC(4)3 anionic dye, indicating the depolarization of plasma membrane. (C) Increase of intracellular Ca^2+^ concentration revealed by the Ca^2+^ indicator Fluo-4 AM already after 20 minutes treatment with α-bisabolol, thus confirming the rapid loss of homeostatic plasma membrane impermeability. Each shown experiment is representative of 5.

To investigate whether the loss of cell viability was related to apoptosis, we stained the BCR-ABL^+^ cells with TO-PRO-3 iodide (which has an elevated affinity for double-strand nucleic acids but does not enter intact plasma membrane) and Annexin V (to determine the phosphatidylserine shift from the inner to the outer leaflet of the plasma membrane). We observed a time-dependent increase of TO-PRO-3 and Annexin V fluorescence when cells were treated with 80 µM α-bisabolol (figure 5A). After 3 hours of incubation with α-bisabolol about 50% of CML-T1 cells were Annexin V^pos^ thus confirming the irreversible onset of the apoptotic cascade. To further assess the damage of plasma membrane after treatment with α-bisabolol, we used the sensitive slow-response probe DiBAC4(3) which enters depolarized cells: increased membrane depolarization results in additional influx of the anyonic dye and an increase in cell fluorescence. The mean fluorescence intensity (MFI) of DiBAC4(3) in CML-T1 cells after 1 and 3 hours incubation with 80 µM α-bisabolol was 3 to 6-fold higher than the control (MFI_1h_ = 18.3±0.8 and MFI_3h_ = 48.7±0.6 vs MFI_basal_ = 7.6±0.8; p<.001), thus indicating the depolarization of plasma membrane, an additional hallmark of the apoptotic process induced by α-bisabolol (figure 5B). Finally, when cells were loaded with the Ca^2+^ indicator Fluo-4 AM, an increase of Ca^2+^ influx was evident already after 20 minutes of incubation with α-bisabolol (figure 5C). These results indicate that α-bisabolol rapidly determines plasma membrane alterations in BCR-ABL^+^ sensitive cells: the loss of plasma membrane integrity is probably the earliest trigger of the apoptotic process induced by α-bisabolol.

### α-bisabolol Causes Loss of Mitochondrial Transmembrane Potential and Increase of Mitochondrial Membrane Permeability in BCR-ABL^+^ Cells

**Figure 6:**
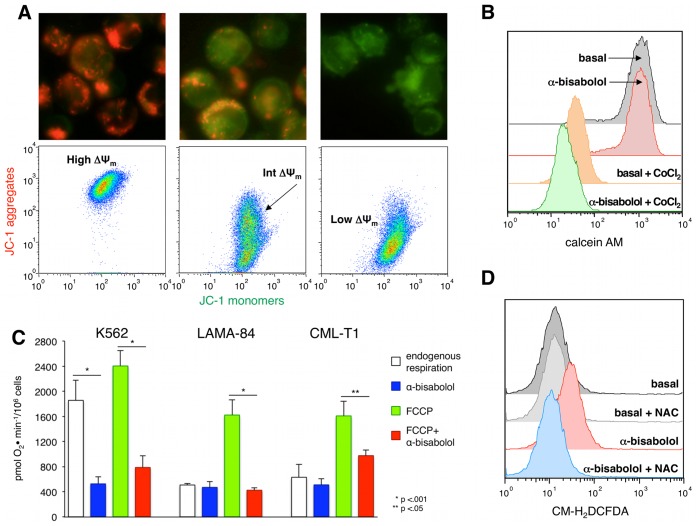
α-bisabolol determined damage to mitochondria in BCR-ABL^+^ cell lines. (A) JC-1 staining demonstrated that α-bisabolol caused dissipation of mitochondrial transmembrane potential (ΔΨ_m_). *Fluorescence microscopy (400x)*. Untreated CML-T1 cells showed intact, well-polarized mitochondria, marked by a red punctate fluorescence; after treatment with 40 µM α-bisabolol for 3 and 5 hours there was a reduction of the red fluorescence and an increase of the green one, indicating loss of ΔΨ_m_. *Flow cytometry*. Untreated CML-T1 cells presented a normal (high) ΔΨ_m_. After treatment with 40 µM α-bisabolol for 3 and 5 hours they moved downward (intermediate and low ΔΨ_m_) because of progressive JC-1 dislocation from mitochondria to the cytosol. (B) Calcein AM assay demonstrated the impairment of mitochondrial permeability transition pore (mPTP) function. After 5 hours of treatment with 40 µM α-bisabolol, CML-T1 cells were loaded with calcein AM: the intensity of fluorescence was similar to that of untreated cells. After adding CoCl_2_ the fluorescence was lower in treated than in untreated cells, indicating that CoCl_2_ entered damaged mitochondria and quenched calcein. (C) Intact BCR-ABL^+^ cells were assayed for oxygen consumption at 30°C in DMEM. Endogenous respiration in presence of FCCP (uncoupled respiration) was significantly inferior in cells treated with 40 µM α-bisabolol for 24 hours than in untreated cells. The results are mean±SD of 3 experiments for each cell line. (D) α-bisabolol induced striking generation of ROS in CML-T1 cells after treatment with 40 µM α-bisabolol for 5 hours. This was completely abrogated by the presence of NAC. Each shown experiment in panels A, B, and D is representative of 5.

In our recent work [13] we demonstrated by JC-1 staining that α-bisabolol dissipates the mitochondrial transmembrane potential ΔΨ_m_) in acute leukemias. Here we investigated the ΔΨ_m_ in BCR-ABL^+^ cells treated with α-bisabolol. CML-T1 cells were incubated up to 5 hours with 40 µM α-bisabolol and stained with JC-1. At microscopy, the fluorescent pattern changed from a punctate red fluorescence (indicating well-polarized mitochondria in untreated cells) to a diffuse green fluorescence (indicating disruption of ΔΨ_m_ in treated cells). At flow cytometry, untreated cells showed well-polarized, red-emitting mitochondria; cells exposed to α-bisabolol lost their red fluorescence, shifting downward over 3 and 5 hours (figure 6A). To further examine the mechanism of mitochondrial function impairment after treatment with α-bisabolol, we used the calcein AM assay. This test explores the activity of mitochondrial permeability transition pore (mPTP), whose opening is an initial event that occurs after cellular damage. Non-fluorescent calcein AM enters cells and become fluorescent after cleavage of AM groups *via* non-specific esterase activity in the cytosol and mitochondria. CoCl_2_ freely passes plasma membrane, but it cannot enter healthy mitochondria, so, when the mPTP is intact (closed), it quenches only cytoplasmic fluorescence thus allowing the detection of mitochondrial fluorescence. CML-T1 cells were incubated for 5 hours with 40 µM α-bisabolol and then loaded with calcein AM: the MFI in treated and untreated cells was similar (1087.5±37.5 vs 1101.5±17.7, respectively, p = ns). After adding CoCl_2_, the fluorescence of treated cells was significantly lower than the fluorescence of untreated cells (19.5±1.6 vs 39.7±4.5, respectively, p<.01), thus indicating a quenching of both cytoplasmic and mitochondrial fluorescence due to the mPTP irreversible opening (figure 6B). These data demonstrate that in BCR-ABL^+^ cells α-bisabolol deeply alters the function of mitochondria, by ΔΨ_m_ dissipation and irreversible disruption of the membrane permeability. So, the apoptotic death of BCR-ABL^+^ cells treated with α-bisabolol depends both by plasma and mitochondrial membrane damage.

### α-bisabolol Inhibits Mitochondrial Respiration in BCR-ABL^+^ Cells

To allow mitochondrial function, endogenous respiration should be coupled to ADP phosphorylation through the trans-membrane electrochemical gradient responsible for the ΔΨ_m_. As shown in figure 6C, treatment of BCR-ABL^+^ cells with 40 µM α-bisabolol for 24 hours resulted in a strong decrease in the oxygen consumption in presence of FCCP (uncoupled respiration) (786±187 vs 2403±244, 423±41 vs 1622±240 and 974±91 vs 1608±234 pmol O_2_•min^−1^/10^6^ cells for K562, LAMA-84 and CML-T1 respectively, p<.05). Endogenous respiration was unaffected by α-bisabolol treatment with the exception of K562 cell line (525±111 vs 1855±324 pmol O_2_•min^−1^/10^6^ cells, p<.001). These data confirm thatα-bisabolol impairs mitochondrial function acting principally on the mitochondrial membrane integrity and determining a detrimental reduction of oxygen consumption that contributes to the apoptotic process.

### α-bisabolol Induces Accumulation of ROS in BCR-ABL^+^ Cells

The production of reactive oxygen species (ROS) is a common feature in apoptotic cells and may indicate the impairment of mitochondrial detoxification. To determine whether α-bisabolol affects the oxydative state of treated cells, we estimated the intracellular concentration of ROS by CM-H_2_DCFDA. This is a cell-permeant indicator for ROS that is nonfluorescent until removal of the acetate groups by intracellular esterases: the intensity of CM-H_2_DCFDA fluorescence is proportional to the cellular amount of ROS. In CML-T1 cells treated with 40 µM α-bisabolol for 5 hours there was a clear increase of fluorescence of CM-H_2_DCFDA loaded cells (MFI_treated_ = 29±2.6 *vs* MFI_basal_ = 11±0.7; p<.01), which was completely abrogated by the addition of N-acetylcysteine (NAC), a scavenger of intracellular ROS (figure 6D). These results indicate that α-bisabolol specifically induces intracellular accumulation of ROS.

## Discussion

This study defined for the first time that α-bisabolol is synergistic with imatinib and nilotinib in a preclinical *in vitro* and *ex vivo* model of BCR-ABL^+^ cells. This synergism with TKI was conclusively measured and it seemed to be relevant in pharmacological terms. A number of α-bisabolol-related biochemical mechanisms that supported the synergism could be revealed and expanded our notions about the selective proapoptotic activity of α-bisabolol against hematologic malignancies.

We have previously characterized the sesquiterpene oil α-bisabolol as an efficient proapoptotic agent with a prefential toxicity to tumor cells, which is probably associated with higher scores of lipid rafts [15] and with the lipophilic properties of α-bisabolol. We showed that, after contact with plasma membrane, α-bisabolol was incorporated into lipid rafts, where it directly interacted with BH3-only Bcl-2 family proteins and that α-bisabolol uptake was higher in transformed glioma cell lines in comparison with non trasformed cells [16]. Therefore it is possible that α-bisabolol induced apoptosis through a preferential accumulation in tumor cells and a selective direct interaction with BH3-only Bcl-2 family proteins such as Bid [14] for example. The efficacy of α-bisabolol was further demonstrated in animal models where it prevented the spontaneous growth of mammary tumors in HER-2 transgenic mice [27] and the growth of subcutaneous and peritoneal pancreas cancer xenografts in nude mice [28].

Recently we tested for the first time the activity of α-bisabolol towards leukemias. We demonstrated that primary acute leukemia cells were sensitive to α-bisabolol at concentrations that did not affect their normal counterpart (i.e. normal CD34^+^ and CD33^+^ myeloid cells). We also observed that α-bisabolol was active against primary BCR-ABL^+^ acute lymphoblastic leukemias (ALL), including cases harboring mutations which conferred resistance to imatinib [13].

In the present study we focused on 3 BCR-ABL^+^ cell lines and 10 cases of *ex vivo* primary BCR-ABL^+^ blasts. This preclinical model allowed us to study the effects of α-bisabolol on BCR-ABL^+^ cells, its mechanisms of action and the combination effects with TKI imatinib and nilotinib.

First, we performed parallel cytotoxic assays on normal PBMC and BCR-ABL^+^ cells: in this way we were able to demonstrate that neoplastic BCR-ABL^+^ cells were significantly more sensitive to α-bisabolol than normal cells (p = .03). Then we evaluated the activity of single agent α-bisabolol in three bone marrow samples of untreated BCR-ABL^+^ ALL with a residual amount of normal T-lymphocytes between 10 and 20%. Also in this case leukemic blast viability was affected by α-bisabolol in a dose-dependent manner while normal cells were relatively spared. We demonstrated that α-bisabolol was preferentially absorbed both into membrane/nuclei and cytosol compartments of malignant cells (p<.001), suggesting that a structural difference between neoplastic and normal plasma membranes could be responsible of the preferential action of α-bisabolol.

α-bisabolol induced several damages to cell membranes. Loss of plasma membrane integrity was clearly demonstrated after 20 minutes of treatment with α-bisabolol. In previous work [16] we demonstrated that α-bisabolol interfered mainly with the mitochondrial membrane integrity rather than directly inhibiting enzymes involved in the mechanism of oxidative phosphorylation. The present work confirmed that the toxic effect of α-bisabolol was due to a strong perturbation of the mitochondrial membranes, indicated by irreversible opening of mPTP which induces not only dissipation of ΔΨ_m_, but also loss of substrates. This lack of matrix substrates is responsible of the decreased respiration rates in presence of FCCP. Therefore α-bisabolol strongly reduced coupled oxygen consumption according to a limited substrates availability. Collectively, these findings supported the notion that α-bisabolol proapoptotic activity mainly depends on induction of intrinsic, mitochondrial-mediated pathway of apoptosis. In addition, a perturbation of cellular homeostasis, as indicated by loss of plasma membrane integrity and increase of Ca^2+^ influx, may contribute to trigger the apoptotic cascade in sensitive cells.

We could demonstrate a full synergism between α-bisabolol and both imatinib and nilotinib. After more than a decade of clinical experience, TKI have demonstrated two main limits: first, about 15% of patients with chronic phase CML and virtually all patients with BCR-ABL^+^ ALL exhibit primary or acquired resistance to imatinib (i.e. they fail to maintain long-term cytogenetic and/or molecular remission); second and perhaps more important, TKI do not eradicate the leukemic stem cell pool even in patients who have an optimal response to TKI, as demonstrated by molecular relapse in the majority of patient who discontinue therapy [3]. These observations have reinforced the concept that BCR-ABL is the main target in CML and BCR-ABL^+^ ALL, but a combination of TKI and novel agents, affecting other cell pathways, might be more effective in preventing the outgrowth of resistant BCR-ABL^+^ cells and targeting the stem cell population. In this view, α-bisabolol appears to be a fascinating candidate. The clear-cut *in vitro* synergism with imatinib and nilotinib strenghtens the notion that α-bisabolol has a proapoptotic activity that is fully independent from BCR-ABL. The demonstration of a specific activity on primary CD34^+^ CD38^−^ BCR-ABL^+^ stem cells is currently under investigation.

Moreover, it has recently been demonstrated that the elimination of BCR-ABL-dependent intracellular signals by TKI causes a sudden decrease of proliferative signals and triggers apoptosis but also activates additional cell survival pathways such as autophagy, in particular in leukemic stem cells. The combination of TKI and autophagy inhibitors resulted highly more efficient in eliminating BCR-ABL^+^ cells, including primary CML stem cells [29]. Apoptosis (type I cell death) is a natural mechanism of tumor repression and neoplastic cells have evolved mechanisms of resistance to proapoptotic signals. Natural or synthetic antitumor agents target this pathways of resistance in order to reset cell sensitivity to apoptosis [30]. An alternative way to induce cell death is autophagy (type II cell death), a regulated mechanism leading to autophagic vacuoles sequestering parts of the cytoplasm and organelles and delivering them to lysosomes for degradation. The relationship between apoptosis and autophagy is complex. Autophagy may also develop as a survival process to adapt to stress condition, suppressing apoptosis. A number of pathways link together apoptosis and autophagy. Cell response may be polarized towards type I or type II cell death and, amongst other molecules, BH3-only Bcl-2 family proteins play a part in this regulation [31]. In this view, α-bisabolol, which may target BH3-only proteins, might both suppress autophagy and induce apoptosis by acting on molecules regulating the switch from autophagy to apoptosis.

In conclusion, we demonstrated in a preclinical model that α-bisabolol was an effective proapoptotic agent against BCR-ABL^+^ cells by targeting several intracellular pathways that determined loss of plasma and mitochondrial membrane integrity. α-bisabolol appeared to be synergistic with imatinib and nilotinib: this may represent the basis for combination of this agent and TKI in the clinical setting, in order to target and eliminate BCR-ABL^+^ stem cells.
